# Student polling software: where cognitive psychology meets educational practice?

**DOI:** 10.3389/fpsyg.2015.00055

**Published:** 2015-01-30

**Authors:** Paul McGivern, Matthew Coxon

**Affiliations:** ^1^Psychology, Department of Life Sciences, College of Life and Natural Sciences, University of DerbyDerby, UK; ^2^Cognitive Psychology Research Group, Faculty of Health and Life Sciences, York St John UniversityYork, UK

**Keywords:** clicker, response system, lecture, attention, memory, vigilance, testing effect

The traditional lecture format still remains one of the most commonly used within higher education, yet it does not provide an optimal environment for learning (Draper and Brown, 2004). Here we will focus upon the use of questioning in large lecture halls and in particular the use of mass audience response systems (also known as clickers or polling systems). In writing this short opinion piece we will bring together some of the key findings from the educational literature, attention literature, memory literature, and wider debates within the Technology Enhanced Learning (TEL) literature, to present an integrated case for the use of online polling software as a partial solution to the challenges of student engagement in lecture halls. To cut a long story short: it is relatively easy to do, students generally like it, and it may well be good for them.

Focused attention is a crucial facet of effective learning (Risko et al., 2013) yet sustaining students' attention in contemporary lecture environments remains a challenge for even the most enthusiastic and engaging lecturers. In practice, maintaining students' attention can involve combatting at least two sources of stimulation competing for attentional resources: mind wandering (e.g., daydreaming, rumination, planning future events) and distraction from mobile technology. These attentional demands must be considered alongside the many challenges of understanding complex unfamiliar material. Mind wandering has been shown to increase with time (as lectures progress) and to impede subsequent comprehension (Risko et al., 2012). The increased presence of technology in lecture environments has also facilitated a rise in students' use of mobile technology devices for non-lecture based activities (e.g., web browsing and social networking) during timetabled sessions (Aguilar-Roca et al., 2012) leading to dual-task learning environments for some students as they divide their attention between the lecture material and different activities. As might be expected, research has shown that engaging in non-lecture based activities on laptops and other electronic devices during lectures negatively impacts on attention and retention. Students who reported high levels of laptop usage (multi-tasking) during lectures correlated with lower course performance and self-reports of a lack of understanding of course materials (Fried, 2008).

One well-established technique for re-engaging attention and minimizing mind wandering is to periodically pose students carefully considered questions. Such questions may test understanding of, or memory for, concepts presented earlier in the lecture or in previous lectures. They may also pose hypothetical scenarios for students to consider, or require them to formulate a view on a certain topic either in groups or individually. However, successfully generating engagement and debate remains challenging given the preceding issues discussed. Resultantly, many universities are now choosing to embrace Audience Response Technologies (ART), also known variously as Audience Response Systems (ARS), clickers, voting systems etc. with emerging research in the area showing that students respond positively to ART reporting that it is easy to use, encourages lecture attendance and is perceived as being both enjoyable and beneficial to their learning (MacGeorge et al., 2008). The interactive element of ART can facilitate wider debate that can subsequently clarify concepts, dispel misconceptions and improve students' understanding of materials (Lundeberg et al., 2011). Additionally, the anonymity provided by ART approaches serves to overcome issues associated with traditional “show of hands” methods, which can lead some students to respond in ways that are consistent with the majority in the room rather than their own thoughts and beliefs. Many students do not feel comfortable participating in lectures by way of more traditional methods; ART therefore offers students a channel of communication to be established between both their lecturer and their peers whilst maintaining a positive learning experience and fostering a more shared learning environment and subsequent participation (Draper and Brown, 2004). Such an approach also resolves limited student responses (often from the same small group of students) that are synonymous with en masse questioning techniques in large lecture theaters, which can subsequently encourage greater mind-wandering among non-participating students.

From a lecturing perspective, use of ART can aid educators in forming a more accurate perspective of how well course materials are being understood via evaluation of polling tools. In addition, students often report the receipt of feedback on their performance as an important facet of their learning (Jump, 2011). Purported increases in attention (and sustained attention) are evident in the findings of Mayer et al. (2009), who reported that students who had been delivered course materials that included ART scored higher on mid-term and final exams, than students receiving materials that contained no ART element. Moreover, students in the ART condition also scored higher than students who were delivered course materials that included a paper-based interactive learning element.

The arguments above assume that there is a relationship between attending to lecture material and subsequent measures of learning and retention. Whilst we might hope such a relationship was causal, the mechanisms that underlie it are unclear and there are likely to be multiple potential explanations for any one individual. Part of any potential explanation may not only include the increased attention to the material but also the experience of being tested on the information. A growing body of evidence suggests that the provision of tests within classrooms does improve subsequent memory for that information, and related information, across a wide range of different types of materials, different types of test, and in different educational settings. In a recent review Dunlosky et al. (2013) explored the effectiveness of 10 different learning techniques that were either drawn from the research literature in cognitive psychology and educational psychology, or were commonly reported by students as a technique they adopted. These therefore ranged from highlighting notes and rereading, to self-explanation and imagery use. Research evidence concerning each of these learning techniques was judged against a set of criteria including the generalizability of the findings to different learner characteristics, and evidence from educational contexts in addition to laboratory settings. In comparing the relative utility, the two techniques that rated most highly were practice testing and distributed practice.

Practice testing generally refers to completing test(s) for information that aren't for summative purposes. The evidence for practice testing is strong, with clear benefits for students across a range of question types which can transfer to improvements in summative assessments, even if presented in different formats (see Glass and Sinha, 2013 for a recent review). In the lecture hall environment, practice testing might involve asking free recall, or multiple-choice, questions of students testing aspects of that lecture, or previous aspects of the course. On courses in which key concepts are carried over from lecture-to-lecture (such as statistics or research methods) repeated testing on these concepts will enable students to better retain and latterly recall these. The use of in-class polls is therefore likely to not only help reinstate attention, but will help latter recall for the information being polled which reflects previously presented lecture content.

The evidence base for the use of in-class polls and voting systems is therefore supported by research literature, within cognitive psychology, which supports both the attentional and memorial benefits to such approaches. As previously stated, the use of ART has begun to be adopted by some universities for different reasons, and we would argue that the attentional and memorial benefits should be part of these. However, staff can often face technical difficulties (e.g., software compatibility, malfunctioning hardware), organizational difficulties (e.g., sourcing funding, technical support) and classroom challenges (e.g., distribution/collection of handsets). One solution to these difficulties is to make use of online polling software such as www.polleverywhere.com.

Online polling software allows students to respond to both multiple-choice and open response questions using a range of technological devices. Results can then be displayed directly in class presentations in different ways. The first clear advantage of online polling over other comparable methods is that students are able to give longer and more detailed responses. Online polling that makes use of devices such as mobile phones allows students to provide text as answers, taking advantage of their own texting skills. This is particularly beneficial in regards to the effects of practice testing, in which free recall of information in response to questions is thought to be more beneficial over and above other methods of testing such as providing cues or “fill in the blank” type answers (e.g., Glover, 1989; Carpenter and Delosh, 2006). More elaborate answers provided in this way also offers the instructor an opportunity to provide more tailored feedback, with informative feedback being an essential part of the learning process (e.g., Bangert-Drowns et al., 1991). The results of online polls are also available to lecturers after the event, allowing them to reflect upon the responses. Furthermore, the use of online polling does not require handsets to be handed out or replaced thus preserving valuable classroom time for learning activities.

In comparison to other uses of classroom technology, online polling software also has the added advantage that it can adequately address the concerns of more conservative adopters of technology and is therefore more viable for wider adoption. It is widely acknowledged that Faculty can often be slow to adopt developing technologies into the classroom and many would argue rightly so. Common concerns about the adoption of technological solutions in classrooms include: increasing divisions between students who do have access to the technology and those that don't; continuous innovation in the absence of an evidence base; and protecting data privacy (Plesch et al., 2013). The use of online polls addresses some of these common concerns that often underpin resistance to adopting technology. Reputable online polls will provide services in which no information about the user is kept or tracked allowing students to engage with the opportunity yet retain their privacy. Online polling that makes use of the text facility of mobile phones, a standard feature of this common technology, would not fuel divisions in the same way that making use of tablet computing, or laptops might. Finally, we hope that the evidence presented here is sufficient for readers to appreciate that whereas the evidence base for many uses of technology is developing slowly, the use of online polling and ART aligns with some of the evidence drawn from cognitive psychology in respect of memory, learning and attention. In addition to the attentional benefits of engaging students with questions, and the memorial benefits of in-class testing, online polling makes use of technology that the vast majority of students have, respects their privacy, and does not require an educator to drastically alter their pedagogical approach as part of the process of adoption.

Maintaining student engagement with lectures against the backdrop of mind-wandering, inattention and technological distraction poses a major challenge for many teaching staff across the sector. Ultimately, permitting students the use of technology in lectures is the lecturer's decision. Allowing the use of technology risks the loss of student attention to non-lecture based technology usage; alternatively, prohibiting technology usage may simply increase the amount of mind wandering. A more palatable alternative to both of these options would be to harness them as lecture-focused technologies as a means of reinstating attention and minimizing mind wandering whilst promoting learning and positively harnessing student preferences for access to/use of technology during lectures. While ART is not a comprehensive “solution” to the aforementioned issues, we have argued here that online polling software is ideally placed to help teaching staff address these challenges. Of course appeals to engage students during lectures, using techniques that help students retain information, and integrating technology in the classroom are by no means new methods. In writing this brief opinion piece our aim was to bring together some of the key findings from educational literature, attention literature, memory literature, and wider debates within the TEL literature, to try and present an integrated case for this single practice. Whilst more evidence is always needed, we are perhaps at a point now where we should be asking why we don't use in-class polling rather than why we do.

## Conflict of interest statement

The authors declare that the research was conducted in the absence of any commercial or financial relationships that could be construed as a potential conflict of interest.
